# 

**Published:** 1885-06

**Authors:** 


### JUNE, 1885.

### CONTENTS.

ORIGINAL PAPERS:— *Page.*

Wounds of the Fingers, and How to Treat Them. By JOHN KENT SPENDER, M.D. Lond. ... 73
Cremation. By J. G. DAVEY, M.D. ... 81
The Border-land of Insanity. By W. B. KESTEVEN, M.D. St. And. 97
A few Remarks upon Aachen (Aix-la-Chapelle). By E. P. PHILPOTS, M.D. ... 104

CLINICAL RECORDS:—

A Case of Hyperpyrexia following Measles, treated successfully by the Wet-pack and Cold Douche. By CHRISTOPHER ELLIOTT, B.A., M.D. ... 109
On Diphtheria: with Cases. By ALFRED SHEEN, M.D. ... 113
Case of Attempted Suicide, by Precipitation from Clifton Suspension Bridge. By J. FENTON EVANS, M.B. ... 124
Rupture of the Aorta. By AUGUSTIN PRICHARD, F.R.C.S. ... 127
Case of Ruptured Aorta. By J. PAUL BUSH ... 129

NOTES ON BOOKS:—

Ebstein's *Regimen in Gout* ... 131
Sir Spencer Wells's *Abdominal Tumours* and *Revival of Ovariotomy* ... 132
*Transactions of the American Surgical Association.* Vol. II. ... 133
Sir Henry Thompson's *Stricture of Urethra* ... 134
Parry's *Extra-Uterine Pregnancy* ... 135
Cotterell's *Common Injuries to Limbs* ... 135
Behrens' *General Botany* ... 135

MEDICAL EXTRACTS ... 136

